# Links between Chinese vocational school students’ perception of parents’ emotional support and school cooperation climate and their academic performance: The mediating role of school belonging

**DOI:** 10.3389/fpsyg.2022.952001

**Published:** 2022-07-29

**Authors:** Yun-Bo Liu, Xiao-Yu Hou, Bin-Bin Chen

**Affiliations:** ^1^Faculty of Education, Beijing Normal University, Beijing, China; ^2^Collaborative Innovation Center of Assessment for Basic Education Quality, Beijing Normal University, Beijing, China; ^3^Department of Psychology, Fudan University, Shanghai, China

**Keywords:** school belonging, academic performance, parental emotional support, school cooperation climate, vocational school students, China

## Abstract

The role of social environmental factors on student academic achievement has been conceptualized from the perspective of the ecological system theory. In the present study, a strengths-based approach derived from the theory of positive youth development was adopted to explore the two favorable aspects of proximal social environments, including parents’ emotional support and school cooperation climate, and to examine how these two factors influence the academic performance among Chinese senior-secondary vocational school students. Participants were 1,940 students (55.4% male) who took part in the Programme for International Student Assessment (PISA) 2018 test from four regions in China. The students completed the questionnaires to assess parents’ emotional support, school cooperation climate, school belonging, and academic performance. By adopting the structural equation model, the results revealed that school belonging fully mediates the association between parents’ emotional support and academic scores, and the association between school cooperation climate and academic scores. In addition, multiple group comparison analyses showed there were some gender differences in the relationships between school cooperation climate and academic performance. The practical significance of the influence of parental support and school cooperation climate on student academic achievement was also discussed.

## Introduction

As the equalizer of social equity, the development of vocational education has been a concern for all sectors of society. Students in China, after 9 years of compulsory education, are generally admitted to secondary vocational schools through the results of the high school entrance examination. Students who attend secondary vocational schools perform worse than those who attend academic senior high schools. In 2020, there were about 16,633,700 students in secondary vocational schools in China, accounting for about 40 percent of the total number of students in senior high schools (Ministry of Education of the People's Republic of China, 2021). According to a survey of Chinese secondary vocational students in 2020, about 65% of 16,900 secondary vocational graduates would continue their studies in institutions of higher learning, which has become the main destination of secondary vocational graduates (Tian, 2022). The academic performance of secondary vocational students deserves attention.

In April 2022, the Chinese government passed an amendment to the Vocational Education Act, highlighting the importance of vocational education to Chinese contribution to global economic and social development. The amendment focuses on were cultivation of quality and student development in vocational education. Studies in western societies (such as Germany, Greece, Italy, Croatia, Austria, and other countries) showed that the academic performance of students in secondary vocational education was often relatively weak (Hanushek and Woessmann, 2006; Kuzmina and Carnoy, 2016). Studies in China showed similar results (Xu, 2015; Loyalka et al., 2016). In terms of the influencing factors of students’ academic performance in secondary vocational education, most of the existing studies focused on the influence of family socioeconomic status (Loyalka et al., 2016) and parents’ educational level (Xu, 2015). Less attention has been paid to social-psychological environmental factors.

The Positive Youth Development Theory emphasizes that socially environmental support is of great significance in adolescent development (Lerner et al., 2005). The positive youth development (PYD) approach moves beyond the negative, deficit focused view of youth (e.g., psychopathology and problem behaviors) and emphasizes the strengths of youth, and their positive qualities and outcomes (Lerner et al., 2009). Adolescents’ positive development is defined as a process in which an individual’s ability to positively interact with the social environment is constantly improved (Guo et al., 2017). In this process, positive social environmental factors play a key role in individual development. Researchers have found that school factors (e.g., school belonging, and group identification at school) and family factors (parent support) both have a positive predictive effect on academic performance based on the frameworks of The Positive Youth Development Theory (Paricio Herrera et al., 2020; Burns et al., 2022). In addition, the environmental factors of academic performance have been conceptualized from the perspective of ecological system theory (Bronfenbrenner, 1979). The theory posits that human development occurs in ecological systems, where the individual interacts with various environments. According to Ecological System Theory (Bronfenbrenner, 1979), the proximal environmental subsystems, such as family and school, play an important role in individual development (Lippert et al., 2019; Gao et al., 2020). School factors and family factors have been found to be closely related to students’ academic performance under the framework of Ecological System Theory (Liu Teng and Zhu, 2019; Kim Sanders et al., 2020). Therefore, this study attempts to explore the impact of family and environmental factors on the academic performance of secondary vocational students in China.

Parents’ emotional support refers to the emotional support parents provide to students in the learning process [Organisation for Economic Cooperation and Development (OECD), 2019] and is a crucial part of students’ family environment. Researchers have found that parents’ emotional support has a positive predictive effect on academic performance among primary school students (Côté et al., 2014), middle school students (Song et al., 2015), and college students (Li et al., 2022). But few studies have explored such relationship patterns among secondary vocational students.

School climate includes the social environment, social interaction, and social norms related to school and learning (Anderson, 1982), including the student–student relationship, the teacher-student relationship, common values, and other dimensions (Lee et al., 2017). School climate has been shown to be related to students’ academic development (Berkowitz, 2021; Demirtas-Zorbaz et al., 2021). School cooperation climate is the evaluation of students for their school cooperation tendency [Organisation for Economic Cooperation and Development (OECD), 2019] and is an important part of school climate. A higher level of school cooperative climate has been shown to be associated with better academic performance of students (Scheerens et al., 2013; Schachner et al., 2021).

School belonging is the feeling of being accepted, respected, included, and supported in the school environment (Goodenow and Grady, 1993). School belonging has been seen as an important factor in academic performance. Some studies focused on the direct impact of students’ school belonging on their academic performance (Korpershoek et al., 2020; Tan et al., 2022), and other research has further explored the mediating role of school belonging on the relationships between family and school factors and students’ academic performance. Studies on the mediating role of school belonging have shown that parent involvement can influence the academic performance of middle and high school students (Kuperminc et al., 2007), that peer cooperation can influence academic performance of American middle school students (Knifsend et al., 2018), and that school climate can influence academic performance among Chinese middle school students (Huang, 2022). However, few studies focus on vocational students.

In addition, researchers have been concerned about gender differences in the academic performance of students (Reilly et al., 2019; Steegh et al., 2019), and research has shown that there are gender differences in the academic performance of vocational education students (Lovat and Darmawan, 2019). Gender is an important moderating variable in studies on the impact of family and school factors on students’ academic performance (Guo et al., 2018). In addition, gender is an important moderating variable in the relationship between school factors and students’ academic performance (Hughes and Coplan, 2018). Therefore, it is necessary to compare gender differences in the relationship between research variables.

Research on the influence of family and environmental factors on students’ academic performance mostly focused on students in basic education and academic-oriented schools, but few on students in vocational education. Some researchers have paid attention to the influence of family socioeconomic status and school conditions (e.g., expenditure level) on students’ math or computer performance (Zhang, 2017; Li et al., 2020), but these studies mostly focused on the influence of material or economic conditions, and have not involved parents’ emotional support, school climate and students’ psychological factors. In addition, some studies on Chinese secondary vocational education students indicated that students’ parents have weak emotional support and students have a weak sense of belonging to school (Liu and Geng, 2018; Huang, 2020). This line of research explored parents’ emotional support and school climate, but did not further examine the impact of these factors on students’ academic performance. These factors are momentous aspects that affect students’ academic performance, and vocational education students are no exception. In this regard, this study focuses on vocational education students in China, expecting to contribute to and enrich vocational education research within the framework of the Theory of Positive Youth Development and the Ecological Systems Theory.

Based on the frameworks of the Ecological Systems Theory and the Theory of Positive Youth Development, the present study used the Programme for International Student Assessment (PISA) 2018 data from four regions in China to investigate the influence of parents’ emotional support and school cooperation climate on the academic performance of secondary vocational students, and explored the mediating role of students’ school belonging. This study also compared the gender differences in the relationship between the variables. The main research hypotheses are as follows:

1. Parents’ emotional support and school cooperation climate would be directly related to the academic performance among secondary vocational students.
2. Parents’ emotional support and school cooperation climate would indirectly influence the academic performance of secondary vocational students through the mediating role of school belonging.
3. Gender would moderate the role of parents’ emotional support and school cooperation climate on the academic performance of secondary vocational students.

## Materials and methods

### Participants

The PISA 2018 test was conducted in four cities in China [Beijing, Shanghai, Jiangsu, and Zhejiang; Organisation for Economic Cooperation and Development (OECD), 2019]. The PISA program is a large-scale international student assessment program conducted by the Organization for Economic Cooperation and Development (OECD) since 2000 to investigate the cognitive abilities of 15-year-old students in reading, mathematics, science, and other aptitudes with mature measurement tools, and high data reliability [Organisation for Economic Cooperation and Development (OECD), 2019]. The full sample included 12,058 students from 361 schools. This study firstly selected secondary vocational students, and obtained 2,088 valid samples of secondary vocational students without outliers. Since the ages of PISA samples were concentrated in 15–16 years old, most students of this age were in the year one of senior high school in China. To ensure the validity, this study sample comprised students in year one of secondary vocational school, totaling 1,940 students (55.4% male). Nearly 50% of the students attended schools in urban areas with a population of more than 1 million. The average family socioeconomic level (ESCS) score for the student sample was −0.82 (the OECD average was 0). Nearly 15% of the students’ parents had received an undergraduate education or above. The percentage of missing variables in the study samples ranged from 0 to 0.4%, and there was no case wherein half the variables were missing. The results of Little’s MCAR test showed that the data met the hypothesis of MCAR [χ^2^ (df = 49) = 52.635, *p* = 0.335]. Missingness was handled *via* full information maximum likelihood in the following analyses.

## Measures

### School belonging

School belonging was measured by the scale of the sense of belonging to school [Organisation for Economic Cooperation and Development (OECD), 2019]. There are three items rated on a 4-point Likert scale ranging from “1” (strongly agree) to “4” (strongly disagree). An example item is “I feel like an outsider (or left out of things) at school.” Cronbach’s α is 0.801.

### Parents’ emotional support

Parents’ emotional support was measured by the scale on the perception of parental emotional support [Organisation for Economic Cooperation and Development (OECD), 2019]. There are three items rated on a 4-point Likert scale ranging from “1” (strongly disagree) to “4” (strongly agree). An example item is “My parents support my educational efforts and achievements.” Cronbach’s α is 0.903.

### School cooperation climate

School cooperation climate was measured by the scale on the perception of cooperation in school [Organisation for Economic Cooperation and Development (OECD), 2019]. There are three items rated on a 4-point Likert scale ranging from “1” (not at all true) to “4” (extremely true). An example item is “It seems that students are cooperating with each other.” Cronbach’s α is 0.935.

### Academic performance

Student academic performance was measured based on reading literacy, mathematics literacy, and science literacy [Organisation for Economic Cooperation and Development (OECD), 2019]. In the PISA 2018 test, all reading, mathematics, and science literacy scores are converted into standardized scores with a mean value of 500 and a standard deviation of 100 based on the sample levels of participating OECD countries. In this study, the first plausible value of students’ reading, mathematics, and science literacy was used for data analysis. Cronbach’s α value of the test scores of the three subjects is 0.910.

### Background variables

The questionnaires of the PISA 2018 include some important demographic variables. Gender and the index of economic, social, and cultural status (ESCS) were selected in this study. Gender is the gender information reported by students. In this study, “1” is male, and “0” is female. The ESCS is a score obtained by factor analysis of family possessions, parents’ education, and parents’ highest occupational status. The mean and standard deviation of ESCS in OECD countries or economies are 0 and 1, and it is an important reflection of students’ social and economic status.

### Data analysis

The data analysis includes three parts. First, we reported the descriptive statistics and correlation results. Second, the structural equation model (SEM) was used to explore whether parents’ emotional support, school cooperation climate, and school belonging were related to students’ academic performance and whether the role of parents’ emotional support and school cooperative climate on students’ academic performance were mediated by school belonging. Finally, the gender differences in the relationship between variables were explored by using the multiple groups comparison analyses.

The SEM was used to analyze the relationship between variables; it is suitable for analyzing the complex relationship with multiple variables and can effectively control the influence of measurement errors. When the root mean square error of approximation (RMSEA) of the model is lower than 0.08 and the comparative fit index (CFI) and the Turkey-Lewis index (TLI) are higher than 0.90, the model is considered a good fit. The standardized factor loadings for any items in the model should be higher than 0.40. Δχ^2^ and Δdf were used in the multiple groups comparison. The data analysis was *via* Mplus 8.3 (Muthén and Muthén, 2017).

## Results

### Descriptive analysis

Descriptive statistics and correlation analysis results of the variables are shown in Table 1. Correlation analysis results showed that there were significant positive correlations among all the variables.

**Table 1:** Descriptive statistics and correlation coefficient.

	M	SD	1	2	3
1. Parents’ emotional support	3.24	0.52			
2. School cooperation climate	2.74	0.68	0.34^***^		
3. School belonging	2.96	0.54	0.36^***^	0.27^***^	
4. Academic performance	527.41	56.47	0.10^***^	0.09^***^	0.13^***^

*** *p < 0.001*.

### SEM analysis

**Figure 1:**
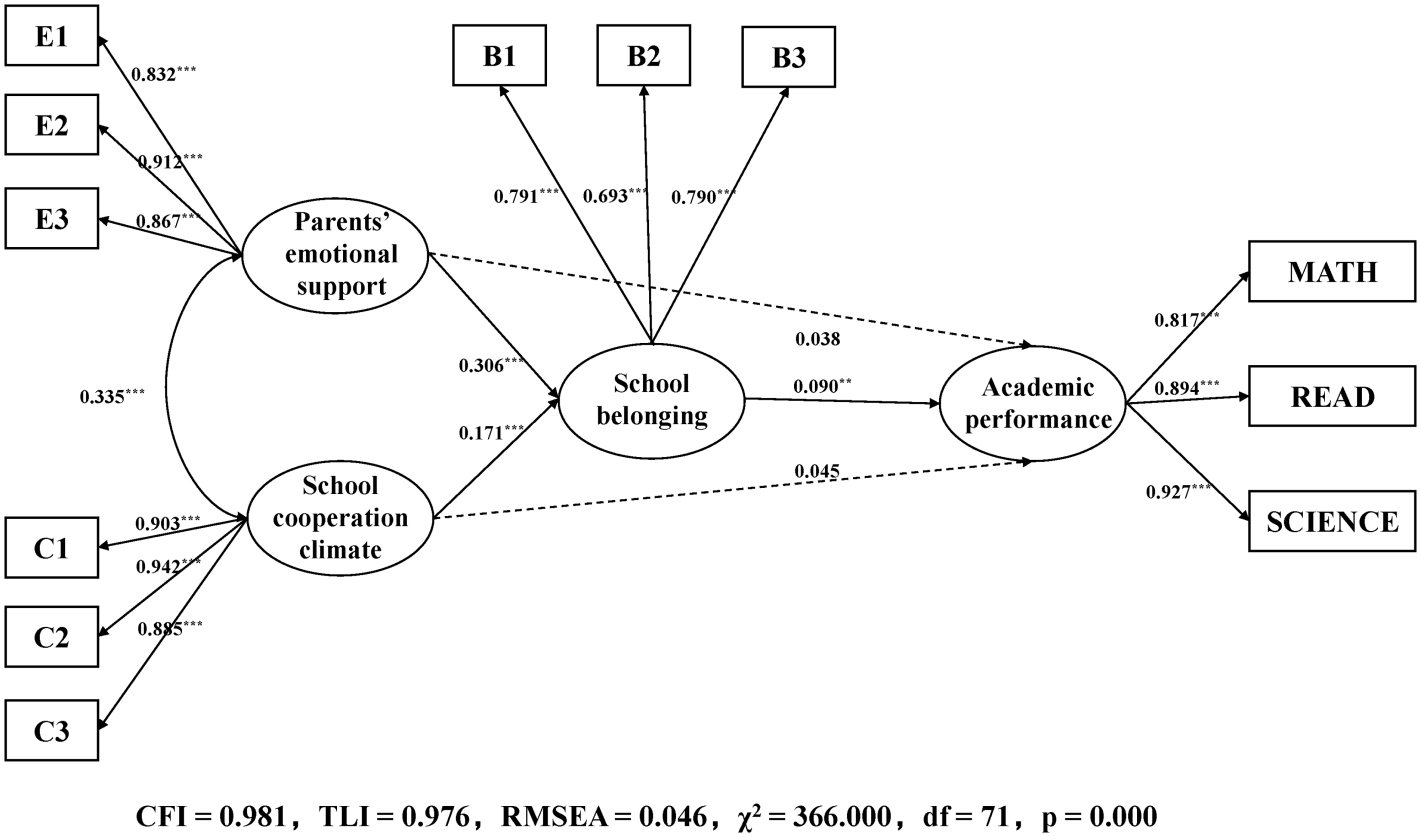
The SEM model with mediating effects (gender and ESCS as controlled variables). ^**^p < 0.01 and ^***^p < 0.001. E1 to E3 mean the three items of perception of parental emotional support; C1 to C3 mean the three items of perception of cooperation in school; and B1 to B3 mean the three items of sense of belonging to school.

The SEM model was used to explore the relationship between variables. The model structure is shown in Figure 1, and the model fits well (CFI = 0.981,TLI = 0.976,RMSEA = 0.046, χ^2^ = 366.000,df = 71,*p* < 0.001). The results of direct effect analysis showed that neither parents’ emotional support nor school cooperative climate had significant predictive effects on academic performance, while school belonging had significant positive predictive effect on academic performance. Parents’ emotional support and school cooperation climate both positively predicted school belonging. Therefore, the first hypothesis (i.e., parents’ emotional support and school cooperation climate would be directly related to the academic performance among secondary vocational students) was not supported.

To test the mediation effects, the bootstrap method was used to test indirect effects, and 1,000x random sampling was used for standard error estimation. The results showed that the effect size of the parents’ emotional support−school belonging−academic performance pathway was 0.028 (*p* < 0.01,95% CI:[0.010, 0.048]), and that parents’ emotional support significantly predicted students’ academic performance through the mediating role of school belonging. The results also showed that the effect size of the school cooperation climate−school belonging−academic performance pathway was 0.015 (*p* < 0.01,95% CI:[0.006, 0.029]), and that school cooperation climate significantly predicted students’ academic performance through the mediating role of school belonging (see Figure 1). Therefore, the second hypothesis (i.e., parents’ emotional support and school cooperation climate) would indirectly influence the academic performance of secondary vocational students through the mediating role of school belonging was supported.

### Multigroup comparison

The gender of students was used as a grouping variable to compare the gender differences. Model 0 is the model in which all path coefficients of the structural model were estimated freely. Model 1 to Model 5, respectively, defined that the relationship between the two groups was equal in parents’ emotional support and academic performance, school cooperation climate and academic performance, school belonging and academic performance, parents’ emotional support and school belonging, and school cooperation climate and school belonging. Model 6 defined that the above five relationship paths were equal. Table 2 shows the comparison between each model and Model 0.

**Table 2:** Multigroup comparison for male and female students.

	χ^2^	df	CFI	TLI	RMSEA	Δχ^2^	*p*
Model 0	455.455	138	0.980	0.977	0.049		
Model 1	456.947	139	0.980	0.977	0.049	1.492	> 0.05
Model 2	460.213	139	0.979	0.977	0.049	5.058	< 0.05
Model 3	455.964	139	0.980	0.977	0.048	0.509	> 0.05
Model 4	458.143	139	0.979	0.977	0.049	2.688	> 0.05
Model 5	455.900	139	0.980	0.977	0.048	0.445	> 0.05
Model 6	463.664	143	0.979	0.977	0.048	8.209	> 0.05

The results showed that there were significant gender differences in the relationship between school cooperation climate and academic performance (Model 2 vs. Model 0,Δχ^2^ = 5.058,Δdf = 1,*p* < 0.05), which indicates that the relationship between school cooperation climate and academic performance of boys and girls are not equal. Specifically, for girls, school cooperation climate had a significant positive effect on academic performance (γ = 0.110,*p* < 0.05), though for boys, school cooperation climate had no significant effect on academic performance (γ = −0.006,*p* > 0.05). In other paths, the model did not become significantly worse if the path coefficients of male and female students were set equally. Therefore, there were no significant gender differences in the relationship between parents’ emotional support and academic performance, between school belonging and academic performance, between parents’ emotional support and school belonging, and between school cooperation climate and school belonging. Therefore, the third hypothesis (i.e., gender would moderate the role of parents’ emotional support and school cooperation climate on the academic performance of secondary vocational students) was partially supported.

## Discussion

The present study is one of the few studies that focus on the relationship between parent’s emotional support, school cooperation climate, school belonging, and students’ academic performance among secondary vocational school students. The findings of the study align with the research hypothesis derived from the Theory of Positive Youth Development and the Ecological System Theory. It was found that both parents’ emotional support and school cooperation climate were positively correlated with students’ perception of school belonging, which in turn affected their academic performance. This implies that students with higher parental emotional involvement and a more cooperative school climate enhances their sense of school belonging and further improves their performance in science, reading, and math. This finding reveals that school belonging fully mediates the association between environmental factors (parents’ emotional support, school cooperation climate, etc.) and students’ academic performance. This is consistent with the findings of other studies that students’ school belonging plays a mediating role in the relationship between school bullying and academic performance (Huang, 2022). A learning environment that offers encouragement and achievement opportunities can enhance the development of a positive self-image, which can result in higher achievement (Mitchell and Conn, 1985; Roeser et al., 1996; Sánchez et al., 2005). Moreover, there was no direct effect of parents’ emotional support and school cooperation climate on students’ academic performance, which highlights the importance of school belonging to students’ academic performance. In terms of the effect size of the indirect effects, parents’ emotional support had a greater effect on secondary students’ academic performance relative to school cooperation climate.

Through an analysis of the above effects on different gender groups, the study found that in terms of the direct effect, school cooperation climate has a significant and positive impact on girls’ academic performance, while it has no such impact on boys’ performance. The result means that girls may benefit more from a positive and cooperative school climate and may face certain challenges due to a competitive school climate. This may be related to difference in socialization development between boys and girls. Girls always exhibit more conformity and obedience traits in school (Ding and Hall, 2007) and are more susceptible to the school climate. In school behaviors, girls show stronger and more persistent engagement compared to boys (Li and Lerner, 2011), which may lead to better performance. In terms of the indirect effect, there is no significant gender difference, and both parents’ emotional support and school cooperation climate positively predict boys’ and girls’ sense of school belonging, which further positively predict their academic performance. These findings may make significant contributions and enrichment to the existing literature on the psychological study of vocational education students.

The findings of the study have many policy implications. In China, most secondary vocational education students are diverted to the vocational track after competitive failures in highly selective academic examinations. For these academically lagging students, parents’ emotional support, a cooperative school climate, and a sense of school belonging are important influences on their academic performance. China is a society that emphasizes academic achievement. Entrance to secondary vocational education has been long considered as failure in academic domains, and children’s poor academic performance may discourage parents. Against this background, education for parents of secondary vocational school students should be strengthened. It is necessary to encourage parents to give more emotional support to children who did not perform well in the past and lead them to believe that parental support can promote their children’s performance in the secondary vocational schools.

Vocational schools may need to launch various types of programs to facilitate cooperation among students and promote a cooperative, win-win campus culture. Effective intervention measures might be taken to enhance students’ sense of school belonging, such as providing curriculum resources including additional classes to help students adapt to life and study in high schools. Considering the gender difference, as Hackett et al. (1992) have mentioned, attention should be paid to the perceived stress and strain of girls, and more encouragement and support should be given by teachers and counselors to help girls make academic progress.

This paper is that it is one of the first to analyze the effect of parents’ emotional support and school cooperation climate on secondary vocational students’ sense of school belonging and their academic performance. The results of the study revealed the importance of school belonging for secondary vocational education students. Previous studies in Belgium and the United States have found that students in vocational schools have lower sense of belongs (Smerdon, 2002; Van Houtte and Van Maele, 2012) and learning involvement (Van Houtte and Stevens, 2009) than academic school students. However, few studies have examined the relations between school belonging and academic achievement among vocational students and its mediating effect. This paper is an enrichment of the Positive Youth Development and the Ecological System Theory, as well as an enrichment of the study of learning in secondary vocational education.

In addition, it also explored the effect of home and school climate on students’ sense of school belonging and emphasized that students’ school belonging not only needs to be enhanced by the school environment, but also by parents who provide a high level of emotional support for their children at home. This can help children develop a sense of security at school outside of the home (Chen, 2017; Chen et al., 2019), which leads to an increased sense of school belonging.

### Limitations

There are several limitations to the current study. Firstly, the study used cross-sectional data to explore the correlations between different factors. Secondly, the findings apply only to secondary vocational schools in eastern China, and whether the conclusions are valid for the central and western regions remain to be verified. The PISA 2018 survey in China only covered four regions, including Beijing, Jiangsu, Zhejiang and Shanghai, where the school facilities and the teaching quality of secondary vocational schools are generally much better than those in central and western China. Thirdly, the academic performance of vocational education students is measured by reading literacy, mathematical literacy and science literacy, which may be not comprehensive enough. In order to reflect the characteristics of vocational education in China, we should add the scores of specialized skills courses to the dependent variables to assess the academic level of vocational education students. Fourthly, the research focused on 15-year-old high school freshmen, and the findings were obtained from this sample. The perceived school climate is significantly different across grades and ages (Ding and Hall, 2007). Sánchez et al. (2005) found that for high school seniors who were considering their future plans, school belonging is less salient in predicting their grades; in contrast, sense of belonging is more likely to play a role in predicting the performance of younger students, such as 1st-or 2nd-year high school students. Their conclusions are consistent with the findings of this paper. Finally, this study was conducted on secondary school students in the context of China’s national conditions and culture. In countries with different cultures and different educational systems, gender plays a different role in predicting the relationship between school environmental factors and students’ academic achievement (Legewie and DiPrete, 2012; Eugene, 2020).

### Future direction

There are several recommendations for the future studies. Firstly, longitudinal data can be used to analyze the casual effect of parents’ emotional support, school climate and school belonging on the academic performance among Chinese vocational students. Secondly, future studies can collect and analyze the data of secondary vocational education students in regions with different levels of economic development and cover vocational schools with different quality levels in China, which can obtain more representative research results. Thirdly, the academic performance of secondary vocational students should not be limited to their academic scores, professional course scores and qualification certificates should also be included in the academic performance, and the academic performance of secondary vocational students can be reflected more comprehensively through multiple indicators. Fourthly, future studies can explore the relationship between the relevant variables of senior secondary vocational students, and the results can also be compared with those of lower grades. Finally, cross-cultural comparative studies based on large-scale international tests can be used to compare differences between countries and cultures in relation to relevant variables in the future.

## Conclusion

In summary, this study adds to the literature about the factors that influence academic performance among Chinese vocational school students. In support of the Ecological Systems Theory and the Theory of Positive Youth Development, this study showed evidence of school belonging as a mediating variable between parents’ emotional support and school cooperation climate and academic performance. These results may enhance our understanding of how positive social environmental factors may be conceptualized in prevention and intervention for improving academic performance among Chinese vocational school students. More work (e.g., additional mediating mechanisms) is needed to better understand the relationships between family and school environments and academic performance among Chinese vocational school students.

## Data availability statement

Publicly available datasets were analyzed in this study. This data can be found at: https://www.oecd.org/pisa/data/2018database/.

## Ethics statement

Given that secondary data was used for the analysis, ethical review and approval was not required for the study on human participants in accordance with the local legislation and institutional requirements. Written informed consent from the patients/ participants or patients/participants legal guardian/next of kin was not required to participate in this study in accordance with the national legislation and the institutional requirements.

## Author contributions

Y-BL and B-BC conceived of the study, developed the hypotheses, motivated the data analyses, oversaw the data analysis, interpreted the results, and drafted and revised the manuscript. X-YH performed the statistical analyses, interpreted the results, and drafted the manuscript. All authors contributed to the article and approved the submitted version.

## Funding

This study was supported by the National Natural Science Foundation of China (grant no. 72104030) and the Humanities and Social Science Fund of Ministry of Education of China, for the project “A Study on the Impact of Input Elements of the Vocational Education on Students’ Non-cognitive Skills - An Empirical Study Based on Value-Added Evaluation” (grant no. 21YJA880040).

## Conflict of interest

The authors declare that the research was conducted in the absence of any commercial or financial relationships that could be construed as a potential conflict of interest.

## Publisher’s note

All claims expressed in this article are solely those of the authors and do not necessarily represent those of their affiliated organizations, or those of the publisher, the editors and the reviewers. Any product that may be evaluated in this article, or claim that may be made by its manufacturer, is not guaranteed or endorsed by the publisher.
